# An Attention-Guided Framework for Explainable Biometric Presentation Attack Detection

**DOI:** 10.3390/s22093365

**Published:** 2022-04-28

**Authors:** Shi Pan, Sanaul Hoque, Farzin Deravi

**Affiliations:** School of Engineering, University of Kent, Canterbury CT2 7NT, UK; psdpluto@gmail.com (S.P.); f.deravi@kent.ac.uk (F.D.)

**Keywords:** biometrics, presentation attack detection, deep learning, Explainable Artificial Intelligence

## Abstract

Despite the high performances achieved using deep learning techniques in biometric systems, the inability to rationalise the decisions reached by such approaches is a significant drawback for the usability and security requirements of many applications. For Facial Biometric Presentation Attack Detection (PAD), deep learning approaches can provide good classification results but cannot answer the questions such as “Why did the system make this decision”? To overcome this limitation, an explainable deep neural architecture for Facial Biometric Presentation Attack Detection is introduced in this paper. Both visual and verbal explanations are produced using the saliency maps from a Grad-CAM approach and the gradient from a Long-Short-Term-Memory (LSTM) network with a modified gate function. These explanations have also been used in the proposed framework as additional information to further improve the classification performance. The proposed framework utilises both spatial and temporal information to help the model focus on anomalous visual characteristics that indicate spoofing attacks. The performance of the proposed approach is evaluated using the CASIA-FA, Replay Attack, MSU-MFSD, and HKBU MARs datasets and indicates the effectiveness of the proposed method for improving performance and producing usable explanations.

## 1. Introduction

Alongside the increasing adoption of biometric technologies, the potential threat of sensor-level spoofing or presentation attacks has also increased rapidly. Facial recognition systems are particularly vulnerable as presentation attack instruments (PAIs) are relatively easy to create and hard to detect. The popularity of social networks (such as Facebook and Instagram) makes high-quality identity-bearing facial information easily available, and biometric information can also be shared at almost no cost. For these reasons, facial spoofing detection research has attracted much attention in recent years [1].

The range and quality of possible PAIs and application environments create particular challenges for PAD. Meanwhile, researchers have dedicated their time in the past two decades to designing robust features for detecting and preventing various presentation attack species. For instance, some feature extractors [2,3] rely on static texture patterns and achieve good results in detecting paper attacks. Many reported works (e.g., [4,5]) favour using temporal information, generally extracted from the sequence of biometric samples. Some approaches adopt a challenge-response strategy, but their requirement for user cooperation may be considered a disadvantage. Alternative approaches (e.g., [5]) using dynamic texture changes have also been proposed, eliminating the need for users’ cooperation. Some recent works [6,7,8,9] using Deep Neural Networks (DNNs) [10] have presented new possibilities for PAD without the need for using “hand-crafted” features. Pre-trained DNN features demonstrate some promising results when evaluated on widely used PAD datasets [11]. However, the opacity of DNN-based approaches may be considered a significant weakness in biometric applications where particular decisions to deny or grant access to individuals must be justified [12]. The question will naturally arise in people’s minds when using these Machine Learning (ML) systems: “Can we trust the decision of this system?”

Explainable Artificial Intelligence (XAI) is an emerging branch of machine learning [12,13,14,15]. In the following discussion, a biometric system, which follows the four principles defined in [15] (i.e., Explanation, Interpretable, Explanation Accuracy, and Knowledge Limit), can be named an Explainable Biometric System. Such systems aim to improve the transparency of current ML algorithms and decrease the opacity of each decision. In recent years, XAI has attracted significant attention and provided some promising solutions [16]. Transparency should be a necessary characteristic for biometric systems due to the need for biometric decisions to be trusted and effectively managed. Rational explanations for the decisions made by a biometric system can help users understand the inner mechanism of decision-making processes. Any questionable decision can be easily identified when the system provides unreasonable justifications. This process thus helps the biometric system build trust with its users rather than impose a decision that may have significant implications for individuals and organisations. Also, understanding the inner mechanism of existing biometric systems may help researchers develop better algorithms. Using the basic theory of XAI, the explanations strengthen the ongoing iterative learning progress and improve overall performance [13]. However, further research is needed to address the interpretability challenge of current automated biometric systems [15,17].

Explainable PAD (X-PAD) ideally aims not only to detect spoofing attacks but also to explain the reasons for its decisions. It could benefit the users by being transparent about the reasons behind its decisions. The explanations produced by X-PAD systems can help with enhancing trust, improving performance, and helping to detect new patterns of security threats. Also, future biometric systems may be required to provide explanations in order to abide by the law [13,15,16,17].

X-PAD systems have various potential users. For instance, in the event of erroneous decisions, system-generated explanations will help the operators identify where the responsibility may lie (similar to flight black-box recorders used for investigations). In some applications, such explanations can avoid mistakes by helping human experts rapidly identify and rectify errors to lower the risk of wrong decisions. Finally, the explanations for the wrong decisions from the current PAD systems can inform researchers to design better systems.

The contributions of this work are four-fold.

- It incorporates the concept of XAI into the PAD task to make the biometric systems more trustworthy. In this work, a DNN framework is introduced which produces human-readable explanations to accompany each decision. The proposed work can justify algorithmic decisions of a DNN-based PAD system using saliency maps and natural languages.
- The justifications for decisions can be tracked and understood and help build trust with users, especially when unexpected rejection or acceptance decisions are produced.
- The framework also learns from these explanations to further improve its own PAD detection accuracy.
- The experiments for evaluating the proposed system use four different benchmarking datasets, which are widely used to evaluate PAD systems. The proposed framework achieves comparable performance concerning other state-of-the-art methods by learning from explanations.

The rest of this paper is organized as follows: In Section 2, a short comprehensive survey is provided for both facial PAD and XAI. The proposed deep neural architecture, which can learn from explanations, is introduced in Section 3. Section 4 shows the experimental details and the performance results for benchmark datasets. Finally, conclusions and possible directions for future work are given in Section 5. Abbreviations includes a list of the abbreviations used in this article. 

## 2. Related Work

Facial presentation attack detection, as a challenging task in biometrics, is reported as a supervised learning problem in the literature [1]. Researchers categorized existing spoofing attacks by defining different presentation attack instruments (PAI): e.g., photographic paper, video projection, or (3D) mask. Meanwhile, each different species of presentation attack can be further divided into sub-categories. For instance, Zhang et al. [18] suggested that paper-based attacks may be categorized by different attack schemes, namely cut-paper attacks and wrapped paper attacks. Chingovska et al. [2] suggested that video attacks may be categorized by screen resolution, screen size, and whether the screen is held by hand. Li et al. [6] showed that the type of camera will also highly affect the result and suggested considering the type of cameras as additional information for training.

To date, several studies have investigated the method of detecting facial presentation attacks. Galbally et al. [4] classified existing works into three groups: Sensor-Level Techniques, Feature–Level Techniques, and Score-Level Techniques. Feature-level techniques, as a low-cost category for this task, have attracted more attention in recent years. Such techniques can be further divided into static and dynamic groups, depending on whether they use temporal information. DNN-based approaches are a sub-category of feature-level techniques which may be distinguished from the conventional feature-based methods (or “shallow features”) [19,20] by their use of trainable convolution layers for biometric feature extraction.

Yang et al. [21] first proposed the use of DNNs for face antispoofing detection. Some works showed that a pre-trained CNN could be transferred to PAD without much fine-tuning [11]. CNNs are effective for face, fingerprint, and iris spoofing detection [8,9,22]. Our proposed framework also utilises a pre-trained CNN by applying a transfer learning paradigm. Additionally, it includes a soft-attention stage [23] and an explainer function to open the “black box” of deep networks for inspection and greater understanding.

Temporal information can also be modelled using DNNs for PAD [7]. For example, by combining a CNN with a Recurrent Neural Network (RNN), Xu et al. [24] proposed architecture to detect various presentation attacks from frame sequences. More recently, CNN has also been extended for spatiotemporal information. Li et al. [6] proposed a 3D CNN-based framework that applies 3 × 3 × 3 convolutions on the video frames for better efficiency and adopts a streamlined strategy for temporal feature learning with different pre-processing and augmentation mechanisms.

Explainable Artificial Intelligence (XAI) for DNNs is an emerging research direction, and there are relatively fewer historical studies in this area. Much of the current literature in this area pays particular attention to defining “what is the explanation”. Visualization of the filters in a CNN, also referred to as perceptive interpretability methods [14,25,26], is one of the direct ways to explore patterns hidden within the neural units. The Up-convolutional network [27] was developed to reverse the feature map back to an image. On the other hand, gradient-based visualization [28] provides a different way of understanding the knowledge hidden within the parameters of a CNN. However, the visual interpretation approaches may generate some meaningless salience maps [25]. There is no commonly used evaluation methodology to quantitatively measure the effectiveness of the visual explanations [26]. The verbal interpretability methods, which can generate explanations using natural languages, could also provide some comprehensible justifications for the decisions [14]. Guo et al. [29] propose a model to provide verbal interpretation for the NLP task. The key problem of verbal interpretability approaches is the model may extract some humanly non-intuitive patterns, or the explanation may not be “clear cut” in their explanations [14]. Recently Brito and Proenca [30] presented a periocular recognition framework that can produce visual explanations. But our proposed work can produce both visual and verbal explanations for the entire face.

In the proposed work, two different approaches are adopted to demonstrate the usability of the explanations using both visual and verbal formats. Providing explanations with both visual form and natural language form allows the proposed system to be more transparent and trustworthy for users. The verbal interpretation helps users appreciate the meaning of the visual salience map. And the visual salience map helps to generate “clear-cut” verbal explanations which focus on the spatio-temporal relations between different filters and objects. Additionally, the explanations are integrated within the proposed algorithm to improve the training of the attention stage resulting in a measurable improvement in detection performance. While recent literature includes papers [15,17,30,31,32] that use explainable AI for biometric recognition, the present work focuses solely on the problem of presentation attack detection for the face modality.

## 3. Methodology

**Figure 1:**
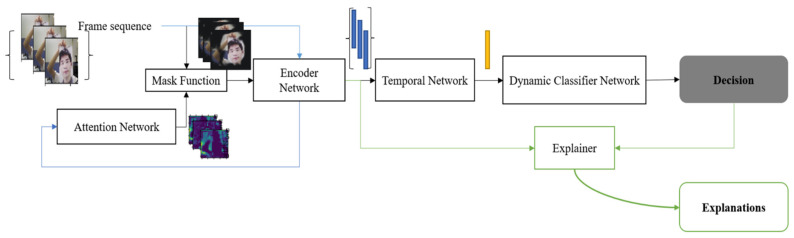
The proposed Dynamic Attention Convolutional Network (DACN). (1) Attention generation (Blue line): Firstly, the frame sequence is fed into the Encoder Network to get the feature representation of each frame. Then the attention maps will be generated by the Attention Network from these original feature representations. (2) Decision generation (Black line): the masked frame, which results from the pixel-wise multiplication of the original frame and the attention mask, is fed into the Encoder Network to get the feature vector for a single frame. Then the Temporal Network is used to encapsulate time-related information. The Dynamic Classifier Network is then used to provide the final decision about the input frame sequence. (3) Explanation generation (Green line): The Explainer function explains current decisions.

This section includes two parts: First, the proposed Explainable PAD (X-PAD) framework. Second, the details about training the proposed framework. We define two variants in the proposed framework of the classifier network, Frame Attention Convolutional Network (FACN) and Dynamic Attention Convolutional Network (DACN), depending on whether or not temporal information is available. The FACN only processes single frames as input, whereas the DACN refers to the pipeline with a Temporal Network. This Temporal Network has been added to process the temporally correlated information and generate a feature vector for the video clips or the frame sequences. Figure 1 shows the DACN version to demonstrate the whole inference pipeline that can handle temporal information.

### 3.1. Proposed Explainable PAD (X-PAD) Framework

The proposed X-PAD system uses DNNs to encode both temporal and spatial texture changes to detect presentation attacks while associating explanations for such decisions. The system can be divided into two functional parts: one is the PAD system which can recognise various facial presentation attacks robustly. Another part is an Explainer that provides some interpretable information for each of the decisions from the PAD system.

Figure 1 illustrates the inference pipeline of the proposed X-PAD system. For X-PAD, the input, denoted by $X=\{X_{i}|i\in \left[1,N\right]\}$*,* is a set of video clips where each clip contains a set of frames $X_{i}=\left\{I_{j}│j\in \left[1,M\right]\right\}$, and the desired output, $Y=\{Y_{i}|i\in \left[1,N\right]\}$, is the set of decisions. The number of decision classes is represented by *C,* which includes genuine presentations and different attack modalities. *N* is the number of video clips in the dataset, and *M* is the number of frames in each clip. Let $\overset{˘}{Y}$ represent the predicted output of the system. The deep learning model, with $\theta_{f},\;\theta_{c}$, as trainable parameters, can be represented by Equation (1):

(1) $$X\overset{F^{f}\left(X;\;\theta_{f}\right)}{\to }E\overset{F^{c}\left(E;\theta_{c}\right)}{\to }\overset{ˇ}{Y}$$

where $E=\left\{E_{i}|i\in \left(0,N\right]\right\}$ is the feature representation of the data generated by the feature extraction sub-network $F^{f}\left(X;\;\theta_{f}\right)$ and $E_{i}=\left\{e_{j}|j\in \left[1,M\right]\right\}$ represents the feature encoding of one video clip. The Encoder Network $F^{f}\left(X;\;\theta_{f}\right)$ and the Classifier Network $F^{c}\left(E;\theta_{c}\right)$ can be designed specifically for PAD and trained from scratch using a PAD dataset. Alternatively, these two sub-networks can also follow the transfer learning paradigm for better generalisation capability. In the proposed experiment, the feature extraction part of a pretrained network based on ImageNet [33] has been transferred for PAD as suggested in [11].

As an X-PAD system, appropriate explanations are created for each decision by feeding the embedded feature vector into the Explainer function *Explain*(.). However, there has yet to be a widely-adopted standard for what could explain a deep learning system. The proposed system uses perceptive interpretation and natural language interpretation for generating human interpretable explanations [13]. The perceptive interpretation can be easily understood by human beings. The proposed framework uses the feature relevance scores calculated by the gradient flow of each decision to measure the influence of spatial importance [28]. The temporal importance, which is also considered a part of the interpretation, is calculated by a modified gate function in LSTM. The interpretations using natural language, which could also be referred to as verbal interpretation, are generated using an NLP method [34].

An additional learning module consisting of an attention network *Attention*($e_{j}$) which has been introduced to improve the performance by an additional learning stage. This module emphasises some locations that may be significantly related to spoofing attacks. The output of the attention network is a prediction of the saliency maps that would be generated by the Explainer function. During training, the explanations calculated by the Explainer function *Explain*(.) are considered as labels for the training of *Attention*($e_{j}$). By integrating this spatial importance map with the original input, the system can focus on the significant regions in each frame. The performance of the proposed X-PAD system can benefit from this step, as will be illustrated in the subsequent experiments.

### 3.2. Training the Attention-Based X-PAD System

There are three stages in the training of the proposed X-PAD system. This section will introduce the different training stages in detail and describes how explanations are used as additional information for improving detection accuracy. It is our contention that an effective X-PAD system could also learn from the explanations generated by itself to improve its performance further. Experiments are designed and conducted to explore this possibility.

**Figure 2:**
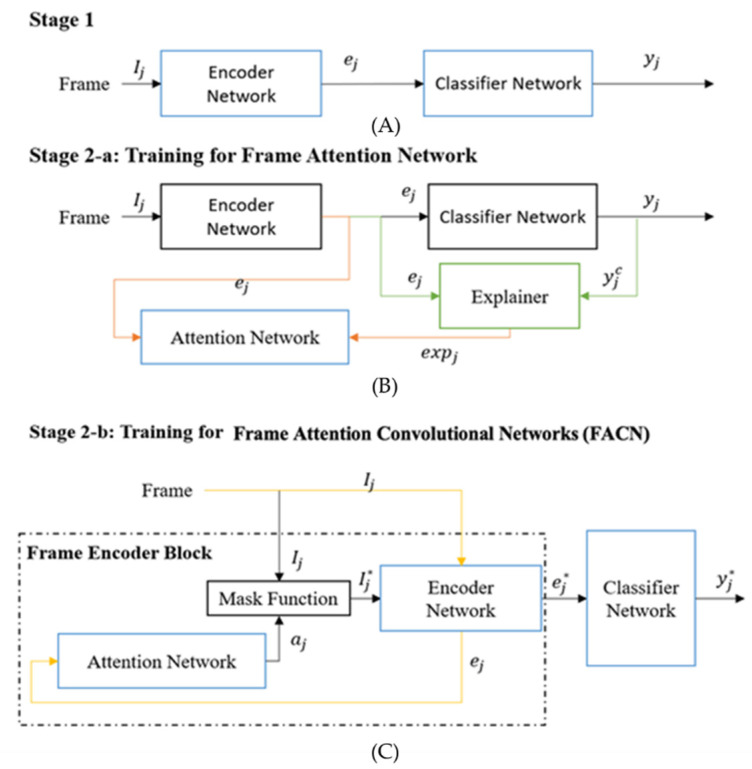
First two training stages: (A) Stage 1, (B) Stage 2-a: Training for Frame Attention Network, (C) Stage 2-b: Training for Frame Attention Convolution Networks (FACN). Blue boxes indicate the sub-network(s) trained in each stage. Stage 1 is a typical DNN architecture for classification. Stage 2 has two phases. In 2a, the Attention Network is trained with the pair of the encoded frame and the spatial explanation from the Explainer function. Then, the Frame Attention Convolutional Network (FACN) is trained end-to-end using new data. The green lines indicate the explanation generation process. The orange lines indicate the training steps to learn with explanations. The yellow line indicates the original frame and the features generated from the original frame.

**Figure 3:**
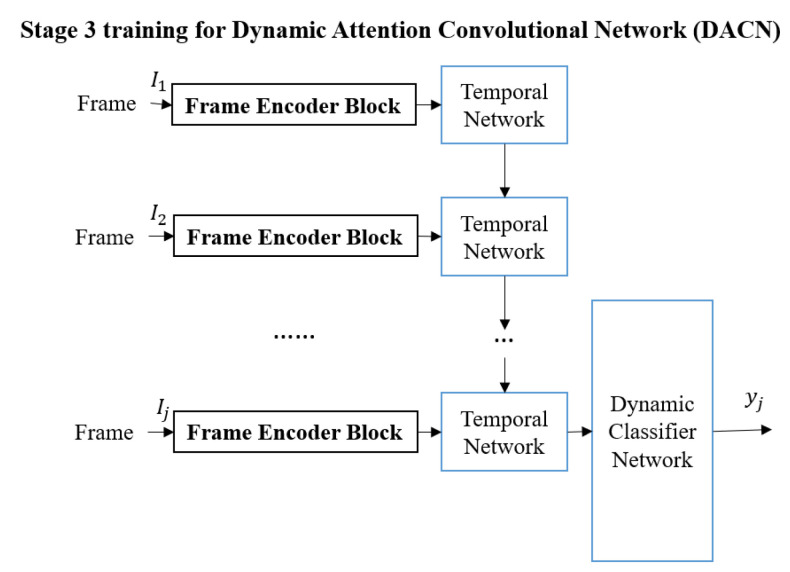
The third training stage: (Blue boxes indicate the sub-network that will be trained.) Stage 3 is used to train the Dynamic Attention Convolutional Network (DACN).

The three training stages for the proposed X-PAD system are illustrated in Figure 2 and Figure 3. The first training stage is a basic DNN learning stage which can also be a transfer learning scheme to adapt a pre-trained convolutional neural network as the Encoder Network to detect facial presentation attacks. The second and the third training stages will help the proposed X-PAD system to produce explanations and learn from them.

The second training stage includes two phases: Stage 2a-Training for the Attention Network, *Attention*($e_{j}$), and Stage-2b Training for the Frame Attention Convolutional Network (FACN). The parameters of the Encoder Network and the Classification Network are shared from Stage 1 and remain fixed in Stage 2a. In Stage 2a, the Attention Network is trained using a dataset that consists of the feature encodings $e_{j}$ for a randomly selected set of frames $I_{j}$ from each video and the related saliency maps generated by the Grad-CAM [28,35]. Every video clip in the training dataset will provide m randomly selected frames for this training where 0 < *m* < *M.* These encoded features are the input of the Attention Network.

The Attention Network $a_{j}$ = *Attention*($e_{j}$) consists of two fully connected dense layers; one with the rectified linear unit (ReLU) activation function [36] and the other with the Tanh activation function. This network produces a spatial importance saliency map for the inference pipeline. When the Attention Network is trained, Stage 2b will commence the training of the FACN. The attention mask $e_{j}$ will be applied to the original frames by using pixel-wise multiplication to get the masked frame $I_{j}^{*}$. Then, the new encoded features $e_{j}^{*}$ are calculated to get the predictions for spoofing attacks. At Stage 2b, the whole FACN is trained end-to-end using a smaller learning rate than that used to train the Attention Network for fine-tuning to improve performance.

The third stage (shown in Figure 3) is used to train the Temporal Network. The deep architecture in Stage 3 is named the Dynamic Attention Convolutional Network (DACN) to emphasize incorporating temporal information. Each video in the training set will be used to train the Temporal Network *Temporal*($a_{j},e_{j}$) which consists of two Long Short Term Memory (LSTM) layers [37,38] to obtain a fixed-length feature for each video. The Temporal Network is used to determine the significant information in the video.

### 3.3. Generating Explanations

The proposed X-PAD system includes two processes: (1) an Explainer block to produce explanations for the current decision and (2) a good learning module to help the system further improve its performance by using these explanations.

Providing explanations for each decision is the key feature of the proposed architecture. The justifications provided by the Explainer function consist of two parts: spatial explanation and temporal explanation. In the proposed system, the Grad-CAM algorithm [28,35] creates a spatial saliency map that indicates the important regions in that frame. To have a better interpretable capability, the proposed method additionally introduces a verbal explanation sub-module to produce natural language explanations. This natural language explanation is generated by using $\xi \left(\overset{ˇ}{Y},exp,Q,L\right)=l$ for the current decision in the proposed work, where *Q* represents a question set and *L* represents the most relevant human language answer set. Here, *l* indicates a natural language expression for the decisions made to accompany the visual explanations *s*. We have provided a set of explanatory expressions in the form of questions and answers, shown in Table 1, as boiler-plate templates to generate the natural language verbal explanations.

The temporal explanations show the most important frame in the video, which may include conclusive evidence for the final decision. The proposed work uses Long-Short-Term-Memory (LSTM) Network [38] to produce temporal-related information. However, the importance of a frame comes not only from the temporal relationship with its neighbours but also from the spatial texture changes. For this reason, we amended the forget gate function of LSTM to $f_{t}^{1}=\sigma_{g}\left(W_{f}e_{t}+U_{f}h_{t-1}+V_{f}^{*}a_{t}+b_{f}\right)$ (the superscript is used to indicate the layer of LSTM) where the $\sigma_{g}$(.) is a sigmoid activation function, $W_{f},U_{f},V_{f}^{*}$ denote the trainable parameters. $h_{t-1}$ is the hidden state of the previous time step and $b_{f}$ is the bias. Here, the attention map $a_{t}$, which is the output of the Attention Network $Attention\left(e_{j}\right)$, is included in the control function *K* of the forget gate. And the cell state function is also changed to integrate input features $e_{t}$ from the Encoder Network, spatial attention heatmap $a_{t}$ and the hidden state of LSTM $h_{t-1}$ as: $K_{t}^{1}=\tan h\left(W_{t}^{1}h_{t-1}+U_{t}^{1}e_{t}+V_{t}^{1}a_{t}+b\right)$. The output of LSTM was fed into a new classifier with two dense layers using the ReLU activation function. 

**Figure 4:**
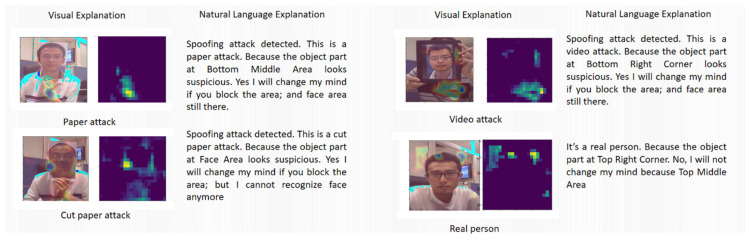
Explanation examples generated by the model for different presentation attack species. In each case, the system provides a saliency map and heatmap as visual justification for the decision and a short paragraph as the natural language explanation (see Table 1 for the list of possible verbal explanations).

The temporal importance explanation is calculated by $exp^{t}=\max\sum \left(f_{t}^{n}+i_{t}^{n}\right)$ to select the time step in which the cell state of LSTM has been maximally changed. In a short frame sequence the proposed method considers the frame, which changes the cell state of LSTM the most, as the most important frame in this sequence. In the proposed method, the spatial saliency map and the temporal importance score guide the training processes in Stages 2 and 3 as additional information. The natural language explanations produced for selected frames can help the human users further understand the reason behind each decision. Examples for both visual and verbal explanations can be found in Figure 4.

## 4. Experiment Design and Results

In this section, we describe the experimental design and implementation details used to evaluate the proposed framework. The results of the experiments are also presented.

### 4.1. Datasets

Four face spoofing detection databases were used in this study for performance evaluations: (1) Replay-Attack [2], (2) CASIA-FA [18], (3) MSU-MFSD [39], and (4) HKBU MARs [40].

The Replay-Attack database includes video clips captured with the front-facing camera of a MacBook. It includes 50 different subjects, and two environmental condition changes are considered when taking the videos. The iPad 1 (1024 × 768 pixels), iPhone 3GS (480 × 320 pixels), and A4 printed paper are used as attack instruments.

The CASIA Face Anti-Spoofing database (CASIA-FA) includes 600 face videos from 50 subjects with different capture quality levels. Paper attacks and video attacks are included in this dataset. The paper attack category consists of warping papers and cut papers as two different categories.

The MSU mobile face spoofing database has 280 videos with 35 subjects, using both a laptop camera (640 × 480 pixels) and an Android phone camera (720 × 480 pixels). Various illumination conditions and subjects with different ethnicities are included for two different presentation attack species (printed photo and video replay attack).

The HKBU MARs Dataset has 120 videos from 8 subjects as a high-quality 3D mask attack dataset, including 2 types of 3D masks (6 from Thatsmyface.com, and 2 from REAL-F). It uses a Logitech C920 web camera (1280 × 720 pixels) to record all the videos with a 25 fps frame rate.

### 4.2. Experimental Setup

Firstly, we used a pre-trained VGG-16 [41] as the Encoder Network. The Classifier Network with two fully connected layers and ReLU activation function is trained using transfer learning in training Stage 1. The Encoder Network (VGG16) is fixed, and the Classifier Network is optimised by using SGD with a learning rate of 0.001. Then, the Encoder Network (VGG16) is fine-tuned but uses a lower learning rate of 10^−7^ at Stage 1. In our implementation, we follow Lucena et al.’s work [11] in fine-tuning the VGG16 network. The Temporal Network includes two stacked LSTM layers (each with 256 hidden units) to learn the important temporal information and the *Attention*($e_{j}$) consists of two dense layers to predict the spatial importance information. The second training stage is optimised using Adam with Cosine Annealing and 100 learning epochs. It is important to note that the VGG-16 network used here is pre-trained on the ImageNet dataset, which is larger than the PAD datasets to be used for the evaluation of the proposed algorithm. This is necessary to avoid the overtraining problem associated with small datasets.

The Grad-CAM [28] algorithm is selected to generate spatial explanations in the proposed framework. In Stage 2, Grad-CAM was also used to provide additional training information for the *Attention*($e_{j}$). As the PAD datasets used in the following experiments do not have pixel-level labels or natural language sentence labels to train a neural network-based natural language generator, we followed Satu et al.’s work [34] to develop a natural language generator in our implementation as in this approach no extra-training data is needed for the natural language generator. In the proposed implementation, the natural language generator selects answers from a pre-defined answer set. The question set and the example answers used can be found in Table 1. Four different questions were included in the question set *Q*. The natural language generator can generate the result *l* by selecting the most relevant answer from result templates *L* using the information from the value of *exp*.

The Replay-Attack database is divided into three subsets: training set, development set, and testing set. The feature encoder network is fine-tuned with 60% of the training set; the *Attention*($e_{j}$) is trained using the rest of the training set. The Equal Error Rate (EER) for the development set is reported and used to determine the threshold to obtain the Half Total Error Rate (HTER) on the test set. For CASIA and MSU databases, the Feature Encoder Network is fine-tuned with 50% of the training set and the *Attention*($e_{j}$) is trained by the rest of the training set. Then, EER is evaluated for the test set following the protocols defined in [12].

### 4.3. Experimental Results

The depth of the Encoder Network is important for performance. In Table 2A, we present the effect of the depth of the Encoder Network using the Replay-Attack and CASIA-FA datasets in terms of Equal Error Rate (EER). There is a clear trend that can be identified; based on the results, deeper networks provide better results. Also, fine-tuning is a useful method to improve the performance of the PAD task. VGG16-block 1-5 and VGG16-block 1-5 (FT) use the same initial network, but the performance difference demonstrates the effectiveness of additional training of the Encoder Network with a fine-tuning (FT) stage.

Table 2B shows that the proposed FACN pipeline can further improve performance by helping the system focus on the important regions. The first 3 rows in Table 2A,B use the same backbone network, but the models that use the proposed FACN pipeline show better performance. Notably, the FACN (block 1-5 FT) nearly halved the EER for both the datasets compared to the best baseline results reported in Table 2A. This effect of the *Attention*($e_{j}$) may be similar to the process of cropping the facial area with the difference that it works at a much finer level focusing on anomalies introduced by the presentation attack. For example, the proposed FACN pipeline is highly sensitive to texture changes in replay attacks (such as moiré patterns). 

Table 2C shows the effectiveness of applying temporal information. The multi-FACN pipeline generates its output for a video clip by averaging the scores for each frame. This is a simple way to integrate temporal information and can be considered as a baseline. The proposed DACN pipeline, on the other hand, exploits the correlation between the frames through the temporal networks and achieves a substantial reduction in EER for the CASIA dataset. It is possible that including the temporal network in the proposed DACN emphasises important frames featuring attack anomalies and reduces the contribution of insignificant frames.

Table 3 compares the performance of the proposed method with selected deep learning methods in spoofing detection. Lucena et al. [11] use the same encoder network as ours and can be considered to provide the performance baseline of Table 3. The proposed workflow uses the same pre-trained feature encoder network as the previously published work [11,20,42]. There is a 58% performance improvement observed for the proposed FACN compared with the single-frame results in [11] for the CASIA dataset, which demonstrates the effectiveness of using the Attention Network *Attention*($e_{j}$). The VGG-16-AD [20] also significantly improves the performance of the pre-trained VGG16 model for the 3D mask attack detection by selecting significant areas within frames. However, their method is only designed for the 3D mask attack detection and performs worse than [11] on the MSU dataset. Secondly, [12,43] also attempts to use temporal and spatial information in their deep architecture. 3DCNN [12] reaches the best result for the Replay-Attack and MSU-MFSD datasets. However, the proposed DACN system achieves the best performance for the CASIA-FASD dataset. Thirdly, a hybrid algorithm is presented in [3], which combines LBP and DNNs. This used to be a popular way to use DNNs which only consider DNNs as a robust feature extractor. However, the proposed method, which consists of only deep neural networks, shows better performance through learning from explanations. These comparisons demonstrate the effectiveness of the proposed approach.

Table 2 shows how the proposed pipeline is instrumental in improving the PAD performance by helping the system focus on the key regions emphasised by the attention saliency map. A human interpretable visual/verbal output also accompanies the PAD outcome. Examples for both visual and verbal explanations can be found in Figure 4. In addition to the fundamental question concerning the nature of the interaction (whether genuine or attack and, if the latter, the attack artifact), the proposed XAI scheme also highlights the key image regions driving its decisions and their influence in the decision process. The spatial saliency maps highlighting the distinctive regions in the test frame are generated by the Grad-CAM algorithm in the *Explainer* block (see Figure 1). The influence of these regions is assessed by filtering out the salient regions in the facial area and checking whether that alters the PAD decision. The verbal explanations are generated by automatically selecting the most appropriate key phrases (from a pre-defined answer set as shown in Table 1). It can be seen that the natural language explanations generated by the system provide an easily understandable summary of the visual saliency results.

To generate visual and verbal explanations, we defined 4 questions for the system to answer (Table-1). The objective here is not just to explain the behaviour of the DL network to technology experts or developers (as in some other XAI papers) but also to give some indications comprehensible to other users (e.g., security system operators). This information can enable these users to quickly highlight whether the system generated a wrong decision or explain the decision to others affected by it. For instance, if a presentation is classified as a spoofing attack (answers to Q1 and Q2, Table 1), the salient region is identified by the system and communicated as the justification for the decision in natural language (answer to Q3). This image region is then occluded, and the attempt is reclassified. If the reclassified image is still detected as an attack, then the decision is confirmed. If a reclassified image generates a different outcome, this is also communicated in a natural language to alert the human operator (answer to Q4). To the best of our knowledge, this approach to Explainable PAD has not been explored before. Additionally, the saliency maps are used to further train the classifier of the PAD system, thus enhancing its performance, as shown in Table 3. Compared to the recently published papers [25,26], the work presented here has the additional advantage of producing human-readable explanations. 

## 5. Conclusions and Future Work

In this paper, we present an explainable face recognition presentation attack detection framework producing both visual and verbal explanations. Grad-CAM saliency maps and the gradient from an LSTM network with a modified gate function are used to produce both human and machine-readable explanations. These are used as additional information to further improve the classification performance. The proposed framework utilises both spatial and temporal information to help the model focus on significant anomalies that indicate spoofing characteristics. The performance of the proposed approach is evaluated using several benchmarking datasets and indicates the effectiveness of the proposed method, improving the detection accuracy by a substantial amount. 

Future work should include evaluations using larger and more challenging datasets, cross-database testing, and unseen attack scenarios. Different pre-trained encoder networks may also be considered in the future (such as ResNet [45] and Inception Net [46]). For mobile applications, the computational efficiency of the encoder network can also be optimised. The Natural Language Processing (NLP) scheme uses conventional techniques in this implementation. Usage of more advanced deep-learning-based techniques may improve performance further. One limitation of the NLP scheme used in this paper is that it needs to be adapted for each unique application, to match the specific requirements of the human users. A more generic solution may be explored in the future. 

## Figures and Tables

**Table 1:** Example of Question and Answering Part.

	Question (Q)	Answer Set (L)	Answer Example (*l*)
1	Is this a spoofing attack?	Spoofing attack detected/It’s a real person	{Spoofing attack detected}
2	What kind of spoofing attack?	Real Face / Paper Attack/ Video Attack/ Mask Attack	This is a {paper attack}
3	Why does the system think this is a spoofing attack?	Face Area/ Top Left Corner/ Top Middle Area/ Top Right Corner/ Left Middle Area/ Right Middle Area/ Centre Area/ Bottom Left Corner/ Bottom Right Corner/ Bottom Middle Area	Because the object part at {face area} looks suspicious
4	If I block that area, will the system change the existing decision?	No, the system will not change the current decision because{}./ Yes the system will change the decision if the user blocks the area; but the system cannot recognize the face anymore/Yes the system will change the decision if the user blocks the area, and the face area is still there	{Yes the system will change the decision if user blocks the area; but the system cannot recognize the face anymore}

**Table 2:** (**A**): Baseline Performance with VGG-16. (**B**): Test results with the proposed FACN. (**C**): Test results with/without the temporal network.

(A)		
	Replay Attack	CASIA
	EER (%)	EER (%)
VGG16-blocks 1-3	25.64	28.71
VGG16-block 1-4	14.73	18.01
VGG16-block 1-5	9.73	10.88
VGG16-block 1-5 (FT)	8.40	9.94
**(B)**		
	**Replay Attack**	**CASIA**
	**EER (%)**	**EER (%)**
FACN (block 1-3 + FT)	12.42	16.84
FACN (block 1-4 + FT)	8.30	9.47
FACN (block 1-5 + FT)	4.45	5.93
**(C)**		
	**Replay Attack**	**CASIA**
	**EER (%)**	**EER (%)**
Multi-FACN	0.20	4.12
DACN	0.37	1.00

**Table 3:** Performance comparison (‘*’ indicates the performance score which follows the reference implemented by ourselves).

Methods	CASIA	Replay-Attack		MSU	HKBU MARs
	EER%	EER%	HTER%	EER%	EER%
VGG16-CNN [11]	9.94 *	8.40 *	4.30 *	5.80 *	28.00 *
VGG-16-AD [20]	-	-	-	6.72 *	11.79
DPCNN [42]	4.5	2.9	6.1	-	-
CNN + LSTM [43]	5.17	3.66 *	4.87 *	7.43 *	31.20 *
LBP-CNN [3]	2.5	0.6	1.3	-	-
3DCNN [12]	1.40	0.30	1.20	0.00	-
DTN [44]	1.34	0.06	0.02	-	-
FACN (Proposed)	4.12	0.20	2.07	1.67	23.70
DACN (Proposed)	1.00	0.37	1.53	0.20	13.51

## Data Availability

Not applicable.

## Abbreviations

CNN	Convolutional Neural Network
DACN	Dynamic Attention Convolutional Network
DNN	Deep Neural Networks
EER	Equal Error Rate
FACN	Frame Attention Convolutional Network
Grad-CAM	Gradient-weighted Class Activation Mapping
HTER	Half Total Error Rates
LBP	Linear Binary Pattern
LSTM	Long Short Term Memory
ML	Machine Learning
NLP	Natural Language Processing
PAD	Presentation Attack Detection
PAI	Presentation Attack Instruments
ReLU	Rectified Linear Unit
RNN	Recurrent Neural Network
SGD	Stochastic Gradient Descent
XAI	Explainable Artificial Intelligence
X-PAD	Explainable PAD
